# Prenatal Exposure to Endocrine Disruptors and Cardiometabolic Risk in Preschoolers: A Systematic Review Based on Cohort Studies

**DOI:** 10.29024/aogh.911

**Published:** 2018-07-27

**Authors:** Daniela S. Gutiérrez-Torres, Albino Barraza-Villarreal, Leticia Hernandez-Cadena, Consuelo Escamilla-Nuñez, Isabelle Romieu

**Affiliations:** 1 Instituto Nacional de Salud Pública (INSP), Cuernavaca, Morelos, MX

## Abstract

**Background:**

Follow-up studies have reported both positive and negative associations between prenatal exposure to endocrine disrupting chemicals (EDCs) and some anthropometric indicators of overweight and obesity in children. However, few studies have evaluated the effect of this exposure on cardiometabolic risk factors in preschool-age children. The health and disease development paradigm (DOHaD) proposes that the physiological and metabolic adaptations triggered by the exposure to these compounds, coupled with postnatal conditions, can modify the risk of disease. In this context, cardiometabolic risk factors in children are not only an important outcome derived from prenatal exposure but a predictor/mediator of the children’s future health.

**Objective:**

To conduct a systematic review of the evidence published in the last 10 years from cohort studies on the association between prenatal exposure to EDCs and cardiometabolic risk factors in preschoolers.

**Design:**

Studies published from January 1, 2007 to May 1, 2017 in PubMed were analyzed. The research strategy was based on specified keywords and following the application of strict inclusion/exclusion criteria, 16 studies were identified and reviewed. Data were extracted and aspects of quality were assessed using an adapted Newcastle–Ottawa scale for cohort studies.

**Results:**

Only 5 of the 16 studies reviewed analyzed cardiometabolic risk factors in addition to anthropometric measures in children. The cohort studies included in this review suggest that prenatal exposure to low concentrations of EDCs has an impact on anthropometric variables and biochemical parameters in preschool-age children. Positive associations between prenatal exposure to EDCs and percentage of fat mass, body mass index, waist circumference, skinfolds and risk of overweight persisted after adjustment for important confounding variables. No association was found with lipid profile and glucose levels.

**Conclusions:**

Evidence was found to suggest that prenatal exposure to EDCs is positively associated with cardiometabolic risk factors in preschool children.

## Introduction

An endocrine disruptor chemical is an exogenous agent that interferes with the production, release, transport, metabolism, binding, action or elimination of natural hormones in the body, responsible for the maintenance of homeostasis, reproduction, development and/or behavior [1]. This heterogeneous group includes phytoestrogens, plasticizing compounds (bisphenol A, phthalates), pesticides, industrial solvents, pharmaceutical agents and potentially toxic metals such as arsenic (As), cadmium (Cd), mercury (Hg) and lead (Pb) [2]. During the first decade after the definition was coined, the investigation of the effects caused by exposure to endocrine disruptors chemicals (EDCs) developed gradually. However, in 2006 Grün and Blumberg [3] proposed that the environmental exposure to EDCs could influence adipogenesis and obesity by increasing the number/size of adipocytes. The so-called “obesogen hypothesis” caused a significant increase in the number of studies designed to assess the effects of this exposure at different moments of life as well as in various health events.

On the other hand, the paradigm of developmental origins of health and disease (DOHaD) establishes that physiological and metabolic adaptations triggered by the exposure to adverse conditions, like undernutrition, infections or chemical exposures, could increase the risk of obesity and the early onset of cardiometabolic risk factors in the offspring [4]. Thus, the presence of cardiometabolic risk factors in children today is not only an important outcome derived from the interaction of prenatal exposures and post-natal conditions, but also is a predictor/mediator of future health.

Follow-up studies have found both positive and negative associations between prenatal exposure to EDCs and some anthropometric variables in children (body mass index, waist circumference, skinfolds, percentage of body fat). However, few studies have evaluated clinical markers of cardiometabolic risk. For this reason, we conducted a systematic review with the aim of analyzing the quality of the evidence published in the last ten years, from cohort studies that have evaluated prenatal exposure to EDCs and the presence of indicators of cardiometabolic risk in preschool children.

## Materials and Methods

### Search strategy and selection criteria

**Figure 1:**
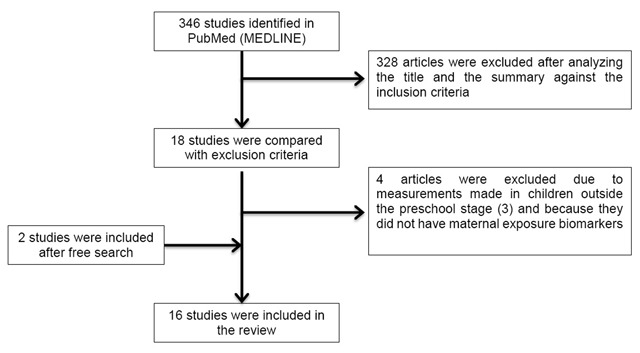
Flowchart for the selection of studies included in the systematic review.

A systematic review of the literature was performed to identify published cohort studies, which investigated the association between prenatal exposure to endocrine disruptors chemicals and cardiometabolic risk markers in the preschool population (3 to 5 years of age). A computerized search of the online databases PubMed (MEDLINE) including all studies published in the last ten years (2007 to 2017) was carried out using the MeSH terms “abdominal obesity”, “body composition”, “hypertension”, “high blood pressure”, “hyperglycemia”, “hypertriglyceridemia”, “cholesterol, hdl”, “cholesterol, ldl”, “HDL/LDL”, “dyslipidemia”, “metabolic syndrome x” and “metabolic syndrome” AND “chemicals, endocrine disrupting”, “metals, heavy” AND “late effects, prenatal exposure”, “maternal exposure” (filters: publication date from 2007/01/01 to 2017/05/01; humans; English, preschool child: 2–5 years) (Figure 1).

### Inclusion criteria

1. Exposure: Any environmental pollutant identified as an endocrine disruptor, regardless of the chemical characteristics of the compound (metals, plasticizers, pesticides etc.) or the human exposure route (inhalation, ingestion, dermal contact etc.).
2. Biomarkers and exposure window: At least one measurement of the biomarkers in maternal biological samples collected during pregnancy (blood, urine, serum or plasma) or at the time of delivery (umbilical cord blood or placenta) regardless of the analytical technique. Exposure recorded at the level of the mother-child binomial.
3. Outcome variable: Clinical report of at least one anthropometric marker or cardiometabolic risk in preschool children who were exposed to EDCs during pregnancy. As anthropometric measurements were considered: height, weight, body mass index, skin folds, waist, abdominal or hip circumference; as well as the indices constructed from these measurements (ex. the waist/height ratio). The diagnosis of overweight/obesity was considered from the cut-off points proposed by the WHO, the CDC or the IOTF, and the markers of cardiometabolic risk of interest were those corresponding to the International Diabetes Federation definition of metabolic syndrome (abdominal obesity, high glucose levels, high triglycerides, low HDL cholesterol levels and high blood pressure).

### Exclusion

1. Studies that reported measurements in children outside the age range of interest (3–5 years).
2. Studies that did not perform prenatal measurements of the exposure.
3. Reviews or systematic reviews, rather than original data.
4. Meeting abstracts, posters, letters or commentaries.

### Data extraction

Data related to the population characteristics, exposure, outcome and adjustment variables were extracted (Table 1). In the case of the studies that reported the outcome variables in continuous scale, the regression coefficients were obtained as a measure of effect. In those that applied cut-off points to generate dichotomous variables, the value of the risk ratio (RR) or odds ratio (OR) with their respective confidence intervals (95% CI) was recorded.

**Table 1:** Characteristics of cohort studies.

Author, location, period of study, follow-up and sample size	Exposure	Outcome	Effect size[β, OR, RR (CI95%)]	Adjustment	NOS

**Percentage (%) of fat mass**					

*Buckley et al. 2016* [11] New York, USA (1998–2002) Follow up: 4 to 9 y (n = 173)	Phenols in maternal urine (3rd trimester of pregnancy).	% of fat mass^a^, BMI^b^ and overweight^e^	*% fat mass (4 y)* 2,5-DCP Boys [β = 0.61 (–1.21, 2.41)], girls [β = 2.94 (0.74, 5.09)] BP-3 Boys [β = –0.02 (–1.73, 1.70)], girls [β = –0.96 (–2.66, 0.75)] BPA Boys [β = 1.42 (–0.35, 3.20)], girls [β = 1.20 (–0.49, 2.85)] Triclosan Boys [β = 0.04 (–1.86, 1.97)], girls [β = 1.01 (–0.55, 2.56)]	Race/maternal ethnicity, age, education, employment, smoking in pregnancy, height and BMI before pregnancy, gestational weight gain, phthalate prenatal exposure (sum of DEHP), lactation, age of the child in months and physical activity in the stage of follow up.	9
*Buckley et al. 2016* [12] New York, USA (1998–2002) Follow up: 4 to 9 y (n = 180)	Phthalates in maternal urine (3rd trimester of pregnancy).	% of fat mass^a^ and BMI^b,c^	*% fat mass (4 to 9 y)* ΣDEHP: T2 vs. T1 [β = –1.77 (–4.48, 0.97)], T3 vs. T1 [β = –3.06 (–5.99, –0.09)] *BMI z-score (4 to 9 y)* ΣDEHP: T2 vs. T1 [β = –0.10 (–0.49, 0.29)], T3 vs. T1 [β = –0.13 (–0.55, –0.29)]	Race/maternal ethnicity, age, education, employment, smoking in pregnancy, height and BMI before pregnancy, gestational weight gain, breastfeeding, age of the child in months and physical activity in the follow-up stage.	8
**Body mass index (BMI)**					

*Maresca et al. 2016* [20] New York, USA (1998–2006) Follow up: 5 to 7 y (n = 337)	Phthalates in maternal urine (3rd trimester of pregnancy) and child urine (3 and 5 years).	BMI^b,c,d^, obesity^f^ and % of fat mass^a^	*BMI z-score (5 to 7 y)* Non DEHP Boys [β = –0.30 (–0.50, –0.10)], girls [β = 0.07 (–0.18, 0.31)] *% fat mass (7 y)* Non DEHP Boys [β = –1.62 (–2.91, –0.34)], girls [β = 0.62 (–0.64, 1.88)]	Race/maternal ethnicity (self-report), public assistance during pregnancy, obesity before pregnancy (self-report), birth weight, age of the child in months at follow-up time and specific urinary gravity (z-score).	7
*Høyer et al. 2015* [17] Greenland and Ukraine “INUENDO” cohort (2002–2004) Follow up: 5 to 9 y (n = 1022)	PFOA and PFOS in maternal plasma (2nd trimester of pregnancy).	BMI^b,c^, overweight^g^ and waist/height ratio (WHtR) > 0.5	*Overweight (5 to 9 y)* PFOA T3 vs.1 Greenland. Boys [RR = 1.03 (0.66, 1.59)], girls [RR = 1.81 (1.04, 3.17)] *WHtR > 0.5 (5 to 9 y)* Pooled analysis PFOA [RR = 1.30 (0.97, 1.74)], PFOS: [RR = 1.38 (1.05, 1.82)].	Maternal age at birth, parity, smoking during pregnancy, education and BMI before pregnancy. The waist/height ratio models were also adjusted for the child’s age and sex.	8
*Tang-Péronard et al. 2014* [21] Faroe Islands (1997–2000) Follow up: 5 to 7 y (n = 561)	PCBs and DDE in maternal serum (3rd trimester of pregnancy) and breast milk.	BMI^b^, overweight and obesity^h^	*BMI at 5 y in girls born of mothers without overweight* PCBs [β = –0.12 (–0.82, 0.58)]; DDE [β = –0.18 (–0.82, 0.45)] *BMI at 5 y in girls born of mothers with overweight* Q4 vs.1 PCBs [β = 0.59 (–0.47, 1.65)]; DDE [β = 0.15 (–0.9, 1.20)]	Parity and maternal age.	8
*Braun et al. 2014* [14] Cincinnati, USA “HOME” cohort (2003–2006) Follow up: 2 to 5 y (n = 297)	BPA in maternal urine (2nd trimester of pregnancy) and child urine (1–2 years).	BMI^b,c^, waist circumference (WC) and overweight^e^	*Change in BMI (2 to 5 y)* Boys [β = 0.0 (–0.5, 0.6)], girls [β = –0.4 (–0.9, 0.2)] *WC* Global [β = –1.5 (–4.0, 1.0)] *Overweight* [OR = 0.65 (0.19, 2.18)]	Maternal race, marital status, parity, age at birth, economic income, education, employment, social security, BMI at 16 weeks of pregnancy, depressive symptoms at baseline and prenatal serum cotinine levels.	8
*Valvi et al. 2013* [24] Spain (2004–2006) Follow up: 4 y (n = 344)	BPA in maternal urine (average of the 1st and 3rd trimesters of pregnancy).	BMI^b,c^, overweight^e^ and waist circumference (WC)	*The prevalence of overweight at 4 years was 21%*. *WC* [β per 10 log units = 0.28 (0.01, 0.57)] *BMI z-score* [β = 0.28 (–0.06, 0.63)] *Overweight* [RR = 1.38 (0.72, 2.67)]	Sex of the child, exact age of event measurement, time of the day in which the woman’s urine sample was collected, country of origin, age at delivery, education, parity, BMI prior to pregnancy and smoking during pregnancy.	7
*Verhulst et al. 2009* [6] Belgium (2002–2004) Follow up: 1 to 3 y (n = 344)	HCB, DDE, PCB and dioxins in umbilical cord blood.	BMI^b,c^	*Difference in the BMI z-score (1 to 3 y)* DDE [β = –0.002 ± 0.001, p = 0.2]; DDE*smoking [β = –0.003 ± 0.001, p = 0.06] PCBs [β = 0.003 ± 0.001, p-value = 0.03] No association with HCB and dioxins was observed	Age of the child, BMI of the parents, maternal age at birth, birth weight in z-score, breastfeeding, maternal smoking before or during pregnancy and household income.	5
**Weight and height**					

*Gardner et al. 2013* [16] Bangladesh (2002–2009) “MINIMat” cohort Follow up: 5 y (n = 1505)	As (weeks 6–8 and 30), Cd (week 8) and Pb (weeks 14 and 30) in maternal urine.	Weight and height^i^	*None of the maternal biomarkers was associated with anthropometric outcomes at 5 years*	SES, chewing tobacco during pregnancy, cooking on fire indoors, maternal education, season of the year in which the birth occurred, parity, sex of the child, anthropometric characteristics and gestational age at birth.	9
**Waist circumference**					

*Philippat et al. 2014* [13] France (2003–2006) “EDEN” cohort Follow up: 1 to 3 y (n = 520)	Phenols in maternal urine (week 22–29 of pregnancy).	Weight, height and waist circumference (WC)	*WC growth rate (mm/week) in children from 1 to 3 years estimated by the increase in 1 IQR* 2,4-DCP β = 2.34 (–0.19, 4.86)], 2,5-DCP [β = 2.18 (–0.91, 5.27)], BPA [β = 0.62 (–2.57, 3.81)], BP-3 [β = 1.18 (–1.36, 3.71)], Triclosan [β = 2.66 (–1.21, 6.53)], Methylparaben [β = 4.18 (0.70, 7.65)], Ethylparaben [β = 1.89 (–1.91, 5.69)] Propylparaben [β = 3.37 (–0.30, 7.04)], Butylparaben [β = 3.61 (–0.43, 7.66)]	Maternal and paternal height, weight before pregnancy, maternal smoking (passive and active) during pregnancy, maternal education, recruitment center, parity and duration of breastfeeding.	6
**Cardiometabolic markers**					

*Vafeiadi et al. 2016* [15] Greece (2007–2009) “Rhea” cohort Follow up: 6 mo to 4 y (n = 235)	BPA free and conjugated in maternal urine (1st trimester of pregnancy) and child urine (3 and 5 years).	BMI^b,c^, obesity^h^, waist circumference (WC), skin folds, blood pressure^j^ and biochemical parameters in blood samples^k^.	*BMI z-score at 4 y* Boys [β = 0.4 (0.005, 0.8)], girls [β = –0.4 (–0.9, 0.05)]. *WC (cm)* Boys [β = 1.3 (–0.7, 3.2)], girls [β = –1.8 (–4.1, 0.5)]. *Skin folds (mm)* Boys [β = 1.9 (–4.0, 7.8)], girls [β = –5.1 (–12.1, 1.9)].	Maternal variables: education, age, BMI before pregnancy, employment and smoking during pregnancy. Variables of the child: birth weight for gestational age, breastfeeding, time to watch television and energy intake at 4 years.	7
*Vafeiadi et al. 2015* [18] Greece (2007–2009) “Rhea” cohort Follow up: 6 mo to 4 y (n = 689)	PCBs, DDE and HCB in maternal serum (1st trimester of pregnancy).	BMI^b,c^, obesity^h^, waist circumference (WC), skinfolds, blood pressure^j^ and biochemical parameters in blood samples^k^.	*BMI z-score at 4 y* HCB: [β = 0.49 (0.12, 0.86)], DDE: [β = 0.27 (0.04, 0.51)] *Obesity* HCB: RR = 8.14 (1.85, 35.81)], DDE: [RR = 3.80 (1.19, 12.14)] *WC > P* HCB: [RR = 3.49 (1.08, 11.28)], DDE: [RR = 3.76 (1.70, 8.30)] *Skin folds* HCB: [β = 7.71 (2.04, 13.39)], DDE: [β = 2.75 (–0.86, 6.35)] *Systolic blood pressure* HCB: [β = 4.34 (0.63, 8.05)], DDE: [β = 2.31 (–0.07, 4.69)] *Diastolic blood pressure* HCB: [β = 2.48 (–0.13, 5.09)], DDE: [β = 1.79 (0.13, 3.46)] *Exposure to PCBs was not associated with obesity or cardiometabolic risk factors in children*	Maternal variables: triglycerides and cholesterol, age, BMI before pregnancy, parity, educational level, smoking during pregnancy. Variables of the child: weight at birth, sex, lactation, gestational age and exact age at the time of measurement.	7
*Tang-Péronard et al. 2015* [7] Faroe Islands (1997–2000) Follow up: 5 y (n = 520)	PCBs and DDE in maternal serum (3rd trimester of pregnancy), HCB in breast milk (4–5 days after delivery).	Insulin and leptin in blood samples^l^	*Insulin > P75 Q4 vs. 1* PCBs: Boys [OR = 1.13 (0.51, 2.48)], girls [OR = 3.74 (1.36, 10.27)] DDE: Boys [OR = 1.56 (0.71, 3.44)], girls [OR = 2.74 (1.08, 6.94)] HCB: Boys [OR = 1.36 (0.80, 2.30)], girls [OR = 1.86 (0.99, 3.47] *Leptin > P75 Q4 vs. 1* PCBs: Boys [OR = 0.78 (0.34, 1.79)], girls [OR = 0.61 (0.24,1.53)] DDE: Boys [OR = 1.01 (0.45, 2.28)], girls [OR = 0.75 (0.31,1.82)] HCB: Boys [OR = 1.25 (0.67, 2.36)], girls [OR = 0.68 (0.34,1.36)]	Maternal age, BMI before pregnancy, parity, BMI of the child at 5 years of age and time of the day in which the blood sample was collected.	8
*Valvi et al. 2015* [19] Spain (2004–2006) Follow up: 4 y (n = 391)	Phthalates in maternal urine (1st and 3rd trimesters of pregnancy).	BMI^b,c^, overweight^e^, waist circumference (WC), waist/height ratio (WHtR) > 0.5, systolic and diastolic blood pressure^m^	*Systolic blood pressure (4 y)* ΣHMWPm Girls T2 vs. 1[β = –0.30(–0.60, –0.01)]; T3 vs. 1[β = –0.32 (–0.62, –0.03)] Boys T2 vs. 1[β = –0.01(–0.34, 0.32)]; T3 vs. 1[β = –0.06 (–0.39, 0.26)]. *No association with WHtR was observed*	Sex of the child, exact age at the time of measurement, characteristics of the mother (country of origin, age at birth, parity, education, social class, pre-pregnancy BMI and smoking in pregnancy).	7
*Kalish et al. 2014* [22] Massachusetts, USA (1999–2002) “Viva” Study Follow up: 4 to 9 y (n = 1031)	Hg in maternal blood (2nd trimester of pregnancy).	Blood pressure^j^	*Prenatal exposure to mercury was not associated with systolic blood pressure in children*	Maternal variables: age, race/ethnicity, education, smoking and prenatal alcohol consumption, fish consumption, marital status, height and weight before pregnancy and history of hypertension. Variables of the child: age and sex.	6
*Hawkesworth et al. 2013* [23] Bangladesh (2002–2009) “MINIMat” cohort Follow up: 4.5 y (n = 1280)	As (week 8 and 30) and Cd (week 8) in maternal urine.	Blood pressure^j^	*Systolic blood pressure (4.5 y)* As [β = 3.69 (0.74, 6.63)], Cd [β = –0.49 (–1.44, 0.45)] *Diastolic blood pressure (4.5 y)* As [β = 2.91 (0.41, 5.42)], Cd [β = –0.21 (–1.02, 0.59)]	Sex of the child, age, socioeconomic index of the parents, height at 4.5 years, season of the year in which the child was born and maternal blood pressure at the beginning of pregnancy.	8

Q: quartile; T: tertile; P: percentile; WC: waist circumference; AC: abdominal circumference; IQR: interquartile range; 2,5-DCP: 2,5 dichlorophenol; BP-3: Benzophenone-3; BPA: Bisphenol A; 2,4-DCP: 2,4 dichlorophenol; DEHP: Di (2-ethylhexyl) phthalate; ΣDEHP: summary measures for DEHP; ΣHMWPm: summary of high-molecular-weight phthalate metabolites; PFOA: perfluorooctanoate; PFOS: perfluorooctane sulfonate; PCBs: polychlorinated biphenyls; DDE: p,p´-dichlorodiphenyldichloroethylene; HCB: hexachlorobenzene; Hg: mercury; As: arsenic; Cd: cadmium. ^a^% fat mass was calculated using the estimates reported by the bioelectrical impedance scale [(fat mass/weight) × 100]. ^b^BMI was calculated as the weight(kg)/height(m^2^) and ^c^was transformed to z-score according to the sex and age of the children. ^e^Overweight=BMI ≥ P85. ^f^Obesity=BMI ≥ P95. ^g^Overweight=BMI>1SD. ^h^Overweight (boys 17.39, girls 17.23) and obesity (boys 19.27, girls 19.2) classification according to the International Obesity Task Force (IOTF) at 5 years of age. ^i^Weight for age and height for age. ^j^Blood pressure was measured with a digital oscillometer. ^k^Biochemical parameters were evaluated in blood samples obtained without fasting. ^l^Insulin and leptin determination was performed using LuminexÒ ^m^Percentiles according to sex, age and height of children.

### Quality assessment

The quality of the publications that were identified for inclusion in the review was assessed using an adapted 9-point Newcastle-Ottawa scale [5] for cohort studies (Table 2). The three parameters considered by this scale are: 1) selection of the study groups (exposed/not exposed), 2) comparability and 3) evaluation of the exposure/event of interest. The maximum score that can be obtained with the NOS scale is 9; however, studies with a score ≥7 were considered high quality for this report. The results of the quality assessment can be found in the last column of Table 1.

**Table 2:** Quality assessment of the cohort studies (adapted from the Newcastle-Ottawa scale). The stars are obtained if the criterion written in italics is met.

**Selection of the study groups**

Representativeness of the exposed cohort: Locally representative of pregnant women. Group of pregnant women with specific characteristics (ex. Women with low socioeconomic status, African-American women). Without description of the selection. Selection of the unexposed cohort: It comes from the same community as the exposed cohort (they share the same risk of being exposed to environmental contaminants during pregnancy). It comes from a different source. Exposure assessment: Two or more measurements taken during pregnancy. Single measurement during pregnancy. Without description.
**Comparability of the groups**

Comparability between groups exposed/not exposed in the design or analysis It was controlled by characteristics of the mother in the statistical analysis. It was controlled by characteristics of the child in the statistical analysis. It was controlled by other environmental exposures in the statistical analysis. It was controlled by concurrent environmental exposures in the preschool age.
**Outcome**

Outcome assessment: Measurement performed directly by personnel standardized in anthropometric methods and/or biochemical tests. Reliable sources independent of the study (medical records). Follow up questionnaire (self-report). The follow-up time was sufficient for outcome to occur: Yes (the age of evaluation in children is >4 years). No (the age of evaluation in children is <4 years). Proportion of the original cohort analyzed The entire cohort was analyzed. >80% of the original cohort was analyzed and/or was described the comparative analysis that proves that the study population is not different from the rest of the cohort. <80% of the original cohort was analyzed. Without information.

## Results

### Description of the included studies

A total of 16 studies fulfilled the selection criteria, and a summary of the characteristics and results of each study is presented in Table 1. Data were collected in the period from 1997 to 2009 and the reports were published between 2009 and 2016. About 30% (5/16) of the studies were conducted in the United States of America and the rest were distributed in the countries of Bangladesh, Belgium, Spain, France, Greece, Greenland, the Faroe Islands and Ukraine. Thirteen of the sixteen studies (81%) were locally representative of pregnant women, only two had regional representation (Prefecture of Heraklion, Greece) and one was targeted to a specific population group (African-American or Dominican women residing in New York).

### Quality assessment

In general, good quality was observed in the studies that resulted from the review. Fifteen of the sixteen (94%) studies received the highest score in quality for the selection and comparability of the study groups. The differences in the final score were mainly due to the variability in the follow-up of the original cohort and the resulting size of the analytical sample (range: 173–1505).

### Exposure measures

The most common biological sample used to measure prenatal exposure to EDCs was urine in the 62.5% of the studies, followed by serum, blood and plasma; only one study collected umbilical cord blood [6]. Regarding the window of exposure, 12.5% of the studies performed a single measurement in the first trimester of pregnancy, 18.8% in the second trimester and 31.3% in the third trimester. Only 25% of the studies evaluated the exposure at the beginning (week 8) and at the end of pregnancy (week 30).

### Outcome measures

The anthropometric outcomes measured in the children were: weight, height, percentage of body fat, skinfolds, body mass index (BMI), abdominal circumference and waist/height ratio. The cardiometabolic risk factors reported were systolic and diastolic blood pressure, total cholesterol, HDL cholesterol, leptin, adiponectin and C-reactive protein. Only one study quantified insulin concentrations [7]. The classification of overweight and obesity in children was made considering the cut-off points of WHO [8], CDC [9] and the international working group on obesity [10].

## Main results by outcome

### 1. Percentage of fat mass

#### Phenolic compounds

Buckley et al. [11] evaluated the association between prenatal exposure to environmental phenols and fat mass in a sample of 173 children between 4 and 9 years of age. In the crude model, the associations between the concentrations of phenols in maternal urine and the percentage of fat mass were positive for 2,5-dichlorophenol (2,5-DCP, β = 1.24, 95% CI: 0.08, 2.40), negative for benzophenone-3 (BP-3, β = –1.13, 95% CI: –2.24, 0.01) and null for BPA and triclosan. After adjustment, biomarkers of phenol exposure were not significantly associated with fat mass. However, a lower percentage of fat mass was observed in girls compared to boys. When performing a sensitivity analysis, they observed an increase in the percentage of fat mass associated with prenatal exposure to 2.5-DCP and BPA in children between 4 and 5.5 years.

#### Phthalates

Buckley et al. [12] analyzed the effect of prenatal exposure to phthalates and found a negative relationship between the sum of metabolites derived from di (2-ethylhexyl) phthalate (ΣDEHP) and the percentage of fat mass in children aged 4 to 9 years old. Compared with tertile 1, the percentage of fat mass in tertiles 2 and 3 was 1.77% (95% CI: –4.48, 0.97%) and 3.06% lower (95% CI: –5.99, –0.09%), respectively.

### 2. Abdominal circumference, weight, height and waist/height ratio

#### Phenolic compounds

In a study conducted in France, Philippat et al. [13] measured prenatal exposure to nine phenolic compounds and evaluated their association with growth parameters in boys of 3 years old. The results showed a positive association between methylparaben and abdominal circumference (β = 4.18, 95% CI: 0.70, 7.65). Following with the phenolic compounds, Braun et al. [14] reported a negative association between prenatal exposure to BPA and waist circumference (β = –1.5, 95% CI: –4.0, 1.0) at 4 and 5 years of age. In contrast, a study conducted in Greece reported a positive association between prenatal exposure to BPA and waist circumference in boys at 4 years (β = 1.3, 95% CI: –0.7, 3.2), while in girls a negative association was observed (β = –1.8; 95% CI: –4.1, 0.5) [15].

#### Metals

Gardner et al. [16] evaluated the effect of early prenatal exposure (week 6 to 14) to arsenic (As), cadmium (Cd) and lead (Pb) on weight and height in children of 5 years old from the MINIMat cohort (Bangladesh). In the unadjusted models, only the exposure to Cd was negatively associated with the anthropometric measures of the children; however, this association disappeared after adjustment.

#### Perfluorinated alkylated compounds

Høyer et al. [17] examined the association between serum concentrations of perfluorooctanate (PFOA) and perfluorooctane sulfonate (PFOS) with the risk of presenting a waist/height ratio (WHtT) > 0.5 in children aged 5 to 9 years in Ukraine and Greenland. The results showed a high overall risk associated with exposure to PFOA and PFOS in both countries and in the overall analysis (n = 1022) the risk of presenting a waist/height ratio greater than 0.5 was 1.30 (95% CI: 0.97, 1.74) and 1.38 (95% CI: 1.05, 1.82) for each increment of a logarithmic unit of PFOA and PFOS in maternal serum, respectively.

#### Polychlorinated biphenyls compounds

Vafeiadi et al. [18] reported that prenatal exposure to DDE and HCB was significantly associated with an increased risk of presenting a waist circumference greater than the 90th percentile (≥58.6 cm) in children of 4 years old (DDE RR = 3.76, 95% CI: 1.70, 8.30, HCB (RR = 3.49, 95% CI: 1.08, 11.28).

### 3. Body mass index and risk of overweight

#### Phthalates

In the study conducted in Greece, Valvi et al. [19] found a negative association between the sum of high molecular weight phthalates (ΣHMWPm) and BMI in boys at 4 years of age (β = –0.38, 95% CI: –0.76, –0.01). These results were replicated by Buckley et al. [12] in a cohort of African American and Dominican women residing in the USA, since a statistically significant association was found between the No-DEHP component and a lower BMI z-score in male children (β = –0.30, 95% CI: –0.50, –0.10, p-value of interaction = 0.003). On the other hand, Maresca et al. [20] analyzed prenatal exposure to phthalates in African-American and Dominican women residing in New York (USA) and the BMI z-score in children aged 5 and 7 years. Their results were similar, showing that exposure to the maternal No-DEHP component was associated with a lower BMI z-score in male children (β = –0.30, 95% CI: –0.50, 0.13).

#### Perfluorinated alkylated compounds

Høyer et al. [17] conducted a study in Greenland and Ukraine to assess the association between prenatal exposure to PFOA and PFOS and the risk of overweight (BMI z-score > 1DS) in children at 5 to 9 years of age. In Greenland, the risk in girls in tertile 3 of exposure to PFOA was 1.81 (95% CI: 1.04, 3.17), and in boys it was 1.03 (95% CI: 0.66, 1.59, p-value of interaction = 0.15). The results in Ukraine were inconsistent; a lower risk was observed in children in the group with the highest exposure (Tertile 3 RR = 0.78, 95% CI: 0.47, 1.29) compared to children of medium exposure (Tertile 2 RR = 1.38 95% CI: 0.91, 2.10). No association was observed with prenatal exposure to PFOS and the risk of overweight.

#### Phenolic compounds

Braun et al. [14] evaluated the effect of prenatal exposure to BPA on the BMI z score in children aged 2 to 5 years. After adjusting, the results showed that the increase in 10 logarithmic units of BPA in maternal urine was associated with a decrease in the risk of being overweight (OR = 0.65, 95% CI: 0.19, 2.18).

#### Polychlorinated biphenyls compounds

Tang-Péronard et al. [21] reported that prenatal exposure to PCB and DDE was not associated with the BMI z-score in children of 5 years of age in the Faroe Islands. In contrast, Verhulst et al. [6] reported that prenatal exposure to PCBs was associated with an increase in BMI of children in the first 3 years of age (β = 0.003, p = 0.03). In this particular study, the maternal history of smoking was considered as an effect modifier variable, and it was observed that in the first year of life, the mean BMI (z-score) in the children born of smoking women was higher in comparison with children of non-smoking women. However, a decrease in this difference was observed as the DDE concentrations in umbilical cord blood increased (10th percentile: 0.68, 90th percentile: 0.18). At 3 years, prenatal exposure to DDE had less effect on the BMI of children of non-smoking mothers (difference between p90 and p10 = 0.13) compared to children of smoking mothers (difference between p90 and p10 = 0.76).

### 4. Indicators of cardiometabolic risk

#### Phenolic compounds

Vafeiadi et al. [15] evaluated the association between BPA exposure in the first trimester of pregnancy and some indicators of cardiometabolic risk in a sample of 235 children from the Rhea cohort (Greece). The results showed that the association between the concentrations of BPA in maternal urine with the BMI z-score and the sum of the skin folds at 4 years of age is sex-dependent: negative in girls (BMI: β = –0.4; 95% CI: –0.9, 0.05; folds: β = –5.1; 95% CI: –12.1, 1.9) and positive in boys (BMI: β = 0.4, 95% CI: 0.005, 0.8, folds: β = 1.9, 95% CI: –4.0, 7.8). No association was found with blood pressure, cholesterol, leptin, adiponectin and C-reactive protein (CRP).

#### Polychlorinated biphenyls compounds

This same group [18] explored the effect of prenatal exposure to PCBs, DDE and hexachlorobenzene (HCB) and cardiometabolic risk indicators in 689 children of the same cohort. The results showed that exposure to HCB was associated with an increase in the BMI z-score (β = 0.49, 95% CI: 0.12, 0.86), an increased risk of obesity (RR = 8.14, 95% CI: 1.85, 35.81) and an increase in the sum of the skin folds (β = 7.71 mm, 95% CI: 2.04, 13.39) at 4 years of age. For DDE, similar results were observed (BMI β = 0.27, 95% CI: 0.04, 0.5, obesity RR = 3.76, 95% CI: 1.70, 8.30). Likewise, exposure to HCB was associated with an increase in systolic blood pressure (β = 4.34 mm Hg, 95% CI: 0.63, 8.05), while exposure to DDE was associated with an increase in diastolic pressure (β = 1.79 mm Hg; 95% CI: 0.13, 3.46). No association was observed with lipid levels.

On the other hand, Tang-Péronard et al. [7] examined whether prenatal exposure to PCBs, DDE and HCB was associated with insulin and leptin levels in children of the Faeroe Islands at the age of 5 years. In girls, the risk of having insulin concentrations greater than the 75th percentile was associated with the highest levels of exposure (quartile 4 vs.1 PCB: OR = 3.74, 95% CI: 1.36, 10.27, DDE: OR = 2.74, 95% CI: 1.08, 6.94; HCB: OR = 1.86; 95% CI: 0.99, 3.47). No association was observed in male children.

#### Metals

Kalish et al. [22] measured exposure to mercury (Hg) in pregnant women in Massachusetts (USA) and blood pressure in children at the age of 3.2 years (n = 1031). In the bivariate model stratified by age, a positive association was observed between Hg exposure and systolic blood pressure in children (Quartile 4 vs. 1: β = 1.3 mmg Hg, 95% CI: –0.5, 3.2).

In the study conducted by Hawkesworth et al. [23], it was observed that prenatal exposure to arsenic in Bangladeshi women was positively associated with blood pressure in children at the age of 4.5 years (β for each increase of 1 mg/L in the average urine PAS concentrations). = 3.69 mm Hg 95% CI: 0.74, 6.63, DBP = 2.91 mm Hg 95% CI: 0.41, 5.42). No association was observed with exposure to Cd.

## Conclusion

The cohort studies included in this review showed that prenatal exposure to low concentrations of EDCs has an impact on the anthropometric variables and biochemical parameters in preschool children. Some of these associations were statistically significant after adjusting for important confounding variables such as maternal BMI, birth weight, breastfeeding, sex of the child, smoking and other environmental exposures, which provides evidence of the participation of environmental exposures in the risk of obesity and cardiometabolic disease.
